# Physical Health Assessments in a Neuropsychiatric In-Patient Setting- an Evaluation of Quality and Compliance

**DOI:** 10.1192/bjo.2026.11670

**Published:** 2026-06-30

**Authors:** Mohammad Faizan Lodhi

**Affiliations:** North Staffordshire Combined Healthcare NHS Trust, Stoke On Trent, United Kingdom

## Abstract

**Aims:**

Performing a comprehensive physical health examination is crucial for individuals admitted to a neuropsychiatric inpatient unit as it helps identify potential underlying organic conditions that may contribute to a patient’s psychiatric presentation. To ensure optimal care, it is essential for the team to prioritize physical health assessments and maintain clear, timely communication to address all health aspects within the recommended time frame.

**Aims**

To improve the quality of physical health assessments performed by clinicians across the wards.

To assess whether physical health assessments being performed met the standards and were carried out within the time frames recommended by the Royal College of Psychiatrists and North Staffordshire Combined Healthcare Trust (NSCHT).

To assess whether physical health assessments were being recorded onto *Lorenzo* electronic patients record (EPR) as recommended by NSCHT.

**Methods:**

As part of the admission clerking process, the clinician is advised to fill out the General Condition Examination form on Lorenzo (EPR) which ensures a complete examination to be carried out within the recommended time frame.

This was a retrospective study using records from patients admitted to Ward 5 of the NSCHT between March 2023 and March 2024, a total number of 44 patients were included in this study. A data collection form was used which included the following parameters:

**Completion status of physical health assessments**

- **Completed** Physical Health Assessment
- **Incomplete** Physical Health Assessment
- **Missing** Physical Health Assessment

**Completion status of General Condition Examination form on** *Lorenzo* **(EPR)**

- Completed physical health assessments and completed General Condition Examination form on *Lorenzo*
- Completed physical health assessments but **no** form on *Lorenzo*
- Data was analysed and graphically represented.

**Results:**

The analysis revealed that 70% of patients underwent complete physical health assessments within the designated timeframe, 14% of the examinations were incomplete/not up to standard and 16% were missing altogether. Reasons for missing assessments were recorded for only 2 patients. These figures fall below the recommended standards.

In relation to the General Condition Examination form on *Loranzo* EPR, only 18% of patients had the form completed, leaving 82% without the recommended documentation.

**Conclusion:**

Figures from the data analyses were found to be below the standards set out by the NSCHT and the Royal College of Psychiatrists. This would suggest potential communication lapses between clinicians and ward staff.

**Key Recommendations:**

- Emphasising the importance of physical health assessments and filling the General Condition examination form as a **fundamental component** in the resident doctor’s induction handbook and induction meetings.
- Adding the procedure of filling out the General Condition Examination form on *Lorenzo* to the induction smartphone application (EOLAS).

